# Correction: Kirste et al. Structural Analysis of Low Defect Ammonothermally Grown GaN Wafers by Borrmann Effect X-ray Topography. *Materials* 2021, *14*, 5472

**DOI:** 10.3390/ma14226969

**Published:** 2021-11-18

**Authors:** Materials Editorial Office

**Affiliations:** MDPI AG, St. Alban-Anlage 66, 4052 Basel, Switzerland; materials@mdpi.com

The Materials Editorial Office would like to make the following change to this paper [1].

1. On page 3: The notation of crystallographic crystal directions and crystal faces needed to be changed. The notation has been corrected in the following paragraphs: “They are overgrown in the lateral $\langle 11\;\bar{2}0\rangle$ directions during many crystallization processes (due to the slender shape of the seed sticks mainly in the $\left[11\;\bar{2}0\right]\;$ and $\left[\bar{1}\bar{1}20\right]$ directions). After reaching the desired lateral size, the crystals are joined in pairs by tiling in order to obtain a larger crystal. To multiply lateral overgrowth and tiling requires continuous mechanical processing of the crystals in the stage of their preparation for a given growth run. The crystals are mainly trimmed and lapped in a proper way. Figure 1 represents a scheme of the multi regrowth of a slender GaN seed and a scheme of combining two newly-grown, already separated from their seeds, crystals by tiling technology. When the combined crystals reach the correct size by growth in the lateral $\langle 11\bar{2}0\rangle$ as well as the vertical $\left[000\bar{1}\right]$ crystallographic directions (growth in the latter direction is not marked), they are used for preparing substrates, shown in Figure 1. As already mentioned, then the wafering procedures are applied.”

2. On page 4: The notation of crystallographic crystal directions and crystal faces in Figure 1 caption needed to be changed. The notation has been corrected in the caption: “Scheme of the substrate manufacturing steps starting from slender GaN seeds to the finished wafer: Step 1, multi regrowth of a slender GaN seed in the lateral $\langle 11\bar{2}0\rangle$ directions; three new-grown crystal areas crystallized in three separated processes are marked; Step 2, new crystal sliced from the overgrown seed of Step 1; Step 3, two cut out crystals are joined together by tiling technology; Step 4, GaN continues to grow in the lateral $\langle 11\bar{2}0\rangle$ directions as well as in vertical $\left[000\bar{1}\right]$ direction in multi regrowth steps; Step 5, when the necessary size is reached the as-grown GaN crystals can be drilled, sliced and polished in a proper way to produce wafers.”

3. On page 6: The notation of crystallographic crystal directions and crystal faces in the sentence “The bars are perpendicular to one of the fastest growth directions, e.g., the $\left(11\bar{2}0\right)$ direction and its opposite direction $\left(\bar{1}\bar{1}20\right)\;$, but initially not to the four symmetrically equivalent $\left(2\bar{1}\bar{1}0\right)\;$, $\left(\bar{2}110\right)$, $\left(1\bar{2}10\right)\;$ and $\left(\bar{1}2\bar{1}0\right)$” should be “The bars are perpendicular to one of the fastest growth directions, e.g., the $\left[11\bar{2}0\right]$ directions and its opposite direction $\left[\bar{1}\bar{1}20\right]$, but initially not to the four symmetrically equivalent $\left[2\bar{1}\bar{1}0\right]$, $\left[\bar{2}110\right]$, $\left[1\bar{2}10\right]$ and $\left[\bar{1}2\bar{1}0\right]$”. The $\left(\bar{1}\bar{1}20\right)$ in the following sentence has been corrected: “While in the first seed enlargement steps growth was limited to only two directions, in the later course seed enlargement typically occurred in all type $\langle 11\bar{2}0\rangle$ directions and areas with ultra-low TDD emerged at the wafer edge in all these six possible directions.”

4. On page 7: The notation of crystallographic crystal directions and crystal faces in the sentence: “In the case of substrate B, six lines of contrast running parallel to the $\left[\bar{1}100\right]$ direction are observed. Four of the contrast lines appear bright and two of the contrast lines appear dark. This effect is caused by a slight bending of the basal plane at the tiling seam as it forms a grain boundary. The overall pattern of line structures can be described as chains of threading dislocation bundles. The main course in the depth of the dislocation bundles is along the 〈0001〉 direction.” has been corrected.

On page 8: The notation of crystallographic crystal directions and crystal faces in the sentence: “This hypothesis is supported by the fact that the end of this defect pattern is limited in the $\left[11\bar{2}0\right]$ and $\left[\bar{1}\bar{1}20\right]$ direction by areas that were formed in a subsequent generation of the crystal growth process.” has been corrected.

On page 10: The notation of crystallographic crystal directions and crystal faces in the sentence: “The growth front of the Am-GaN crystal proceeded from right to left in $\left[11\bar{2}0\right]$ direction.” has been corrected. The notation in the sentence: “It should be kept in mind that not only in the lateral direction, but also in the vertical $\left[000\bar{1}\right]$ growth direction, phenomena such as growth bands and striations are typically present in ammonothermally grown GaN due to the regrowth steps, as was already shown by Sintonen et al. [39]. Another observation that can be made in the section of the two topographs (Figure 9) is that TDD gradually decreases in the lateral $\left[11\bar{2}0\right]$ growth direction; to the right of the growth band TDD is the highest, in the growth band area itself there is a medium TDD and to the left of the growth band TDD is lowest.” has also been corrected.

On page 11: The notation of crystallographic crystal directions and crystal faces in the sentences: “These dislocations appear like a network forming a continuous wall that extends perpendicularly to the $\left[\bar{1}2\bar{1}0\right]$.”; “Again, as with the example before (Figure 9), separated areas of distinct TDD can be observed for the different lateral regrowth areas with the lowest TDD in the regrowth area at the outer edge in the $\left[\bar{1}2\bar{1}0\right]$ direction”; and “The DB chains always run along an *a*-plane $\left\{11\bar{2}0\right\}$ growth facet.” has been corrected.

On page 12: The notation of crystallographic crystal directions and crystal faces in the sentence: “The outer edges of the hexagons always run along the *a*-plane $\left\{11\bar{2}0\right\}$ prismatic facets and these clusters can reach the size of more than 1 mm edge length for the wafers investigated here (and more than 2 mm edge length for substrates examined in other studies).” has been corrected.

On page 13: The notation of crystallographic crystal directions and crystal faces in the sentence: “The single and chain-like DBs are defects that are manifested in the growth steps of seed crystal enlargement and always arrange themselves along *a*-plane $\left\{11\bar{2}0\right\}$ facets. It is shown that these DBs arise not only at the interface between two regions of different regrowth steps (as in the example of a DB chain visible in Figure 12b), but they also can arise suddenly in the middle of a seed enlargement growth phase (as in all examples shown for single DBs (Figure 11) and the DB chain of Figure 12b). The formation point of the DBs with honeycomb structure (Figure 13) is currently less clear. It could be during the growth for seed crystal enlargement along *a*-plane $\left\{11\bar{2}0\right\}$ facets or at the growth in the vertical $\left[000\bar{1}\right]$ directions.” has been corrected.

On page 15: The notation of crystallographic crystal directions and crystal faces in the sentence: “It should be remarked that a 1–2 orders of magnitude lower TD is found in the most recent grown areas in the lateral $\langle 11\bar{2}0\rangle$ direction.” has been corrected.

We apologize for any inconvenience caused to the readers by these changes. The changes do not affect the scientific results.
